# Chitosan-mediated nano-bioprocessing of *Acacia seyal* seed extract for enhanced antineoplastic, anti-*Helicobacter pylori* and antioxidant performance

**DOI:** 10.1186/s40643-026-01031-6

**Published:** 2026-04-02

**Authors:** Aisha M. H. Al-Rajhi, Marwa Yousry A. Mohamed, Areej Mothana, Abdulaziz Debaji, Magdah Ganash, Yahya Ali, Asmaa A. Alharbi, Tarek M. Abdelghany

**Affiliations:** 1 Department of Biology, College of Science, Princess Nourah bint Abdulrahman University, P.O. Box 84428, 11671 Riyadh, Saudi Arabia; 2 Department of Biology, College of Science, Imam Mohammad Ibn Saud Islamic University (IMSIU), P.O.Box 90950, Riyadh 11623,, Saudi Arabia; 3 Pharmacy Department, Jazan University Hospital, Jazan University, Jazan, Saudi Arabia; 4 Department of Laboratories, College of Nursing and Health Sciences, Jazan University, Jazan, Saudi Arabia; 5 Department of Biological Sciences, Faculty of Science, King Abdulaziz University, 21589 Jeddah, Saudi Arabia; 6 Department of Biology, College of Science, Jazan University, P.O. Box 114, Jazan 45142,, Saudi Arabia; 7 Botany and Microbiology Department, Faculty of Science, Al-Azhar University, Cairo, 11725 Egypt

**Keywords:** *Acacia seyal*, Methyl gallate, Chitosan nanoparticles, HCT116 cells, *Helicobacter pylori*

## Abstract

**Abstract:**

*Acacia seyal* is a medicinal plant rich in bioactive compounds known for their antimicrobial, antioxidant, and anticancer properties. Conventional plant extracts often suffer from poor stability and limited bioavailability, reducing their therapeutic effectiveness. The utilization of chitosan nanoparticles (CS-NPs) offers a promising strategy to enhance biological activity of plant extracts. Encapsulation of *A. seyal* seeds extract (ASSE) in CS-NPs (ASSE@CS-NPs) was the aim of the present investigation to compete *Helicobacter pylori* and HCT116 cancer cells. The phytochemical profile of ASSE revealed diverse phenolic acids and flavonoids via HPLC analysis. Methyl gallate was dominated (20.98 mg/g), followed by gallic acid, and catechin. FTIR analysis confirmed characteristic functional groups in ASSE and CS-NPs. Peak shifts and intensity variations in ASSE@CS-NPs indicated successful encapsulation through hydrogen bonding and electrostatic interactions. ASSE@CS-NPs showed the strongest anti-*H. pylori* effect with inhibition zone (23.7 mm) and lowest MIC/MBC (15.62 µg/mL). Compared to CS-NPs (20.7 mm, 31.25 µg/mL) and ASSE (19.7 mm, 31.25 µg/mL). ASSE@CS-NPs showed notable antioxidant activity, achieving 96% scavenging at 1000 µg/mL with IC_50_ of 3µg/mL compared to ASSE (5 µg/mL) and CS-NPs (74 µg/mL). ASSE@CS-NPs exhibited potent cytotoxicity against HCT116 cells with IC_50_ of 23 µg/mL, outperforming ASSE (588 µg/mL) and CS-NPs (120 µg/mL). The formulation achieved > 80% inhibition at 62.5 µg/mL. Molecular docking was performed to evaluate the binding affinity and interaction patterns of methyl gallate (a main constituent of ASSE) and chitosan against *H. pylori* urease (PDB ID: 6ZJA). Docking results revealed favorable binding scores for both ligands, with chitosan showing higher affinity (S score: − 6.42 kcal/mol) compared to methyl gallate (S score: − 5.45 kcal/mol). Key hydrogen bond interactions were observed with active site residues ASP362 and ALA169 for methyl gallate, and ASP223 and HIS323 for chitosan. These results suggest that ASSE@CS-NPs possess inhibitory potential against *H. pylori* and HCT116 cells.

**Graphical abstract:**

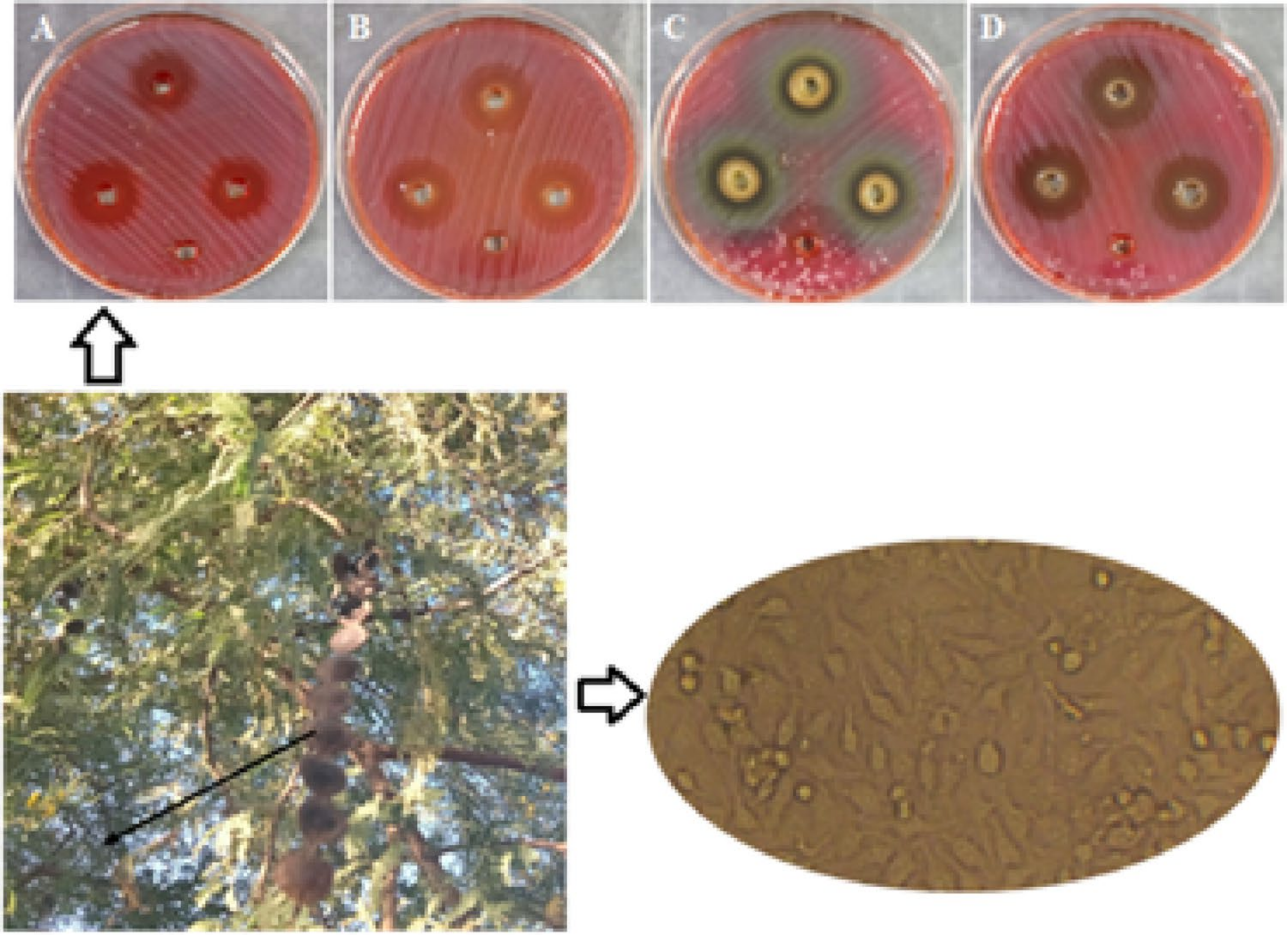

**Supplementary Information:**

The online version contains supplementary material available at 10.1186/s40643-026-01031-6.

## Introduction

Nanoscience still receives great attention from many scientists for serving various fields related to medicine, agriculture, and the environment (Abdelghany et al. 2020; Alsolami et al. 2025). Moreover, scientists are constantly working to develop this field. Previous studies have succeeded in synthesizing many elements in nano form or converting numerous polymer compounds into their nanoscale form. Scientists have also sought to enhance the effectiveness of plant extracts by loading or incorporating them into nanomaterials (Al-Rajhi and Abdelghany 2023a**).** The search for unique and relatively new bioactive chemicals of natural origin has increased in recent years due to their wide range of applications in the food, healthcare, and biomedical industries. Naturally derived compounds like carminic acid and mangiferin have received a lot of attention due to their various biological activities, which include antioxidant, antibacterial, anti-inflammatory, and anticancer effects (Castro-Muñoz et al. 2024). Comprehensive research has identified advanced extraction routes and purification strategies for carminic acid, stressing its promise as both a natural food colorant and a physiologically active molecule appropriate for inclusion into delivery systems. Similarly, mangiferin has received substantial attention for its multifunctional biologic properties and potential for encapsulation and tailored delivery uses in food and pharmaceutical applications (Castro-Muñoz et al. 2024; Ferreyra-Suarez et al. 2024).

Wild populations of medicinal plants are recognized as valuable reservoirs of naturally occurring compounds with potential biological and pharmacological effects (Alawlaqi et al. 2023; Al-Rajhi and Abdelghany 2023b; Al-Rajhi et al. 2024; Alsalamah et al. 2025a). Among them, the genus *Acacia* (commonly known as wattle), a member of the Fabaceae family, encompasses more than 1,350 woody species distributed predominantly across warm climates in regions such as Australia, the Americas, Africa, and Asia (Adiamo et al. 2020). Numerous studies have explored the bioactivity of *Acacia* extracts, particularly from flowers and leaves, with a focus on *A. saligna*. In Egypt, leaf extracts of *A. nilotica* and *A. seyal* have demonstrated stronger antioxidant properties compared to those of *A. laeta* (Abdel-Farid et al. 2014). Research on the aerial parts of several species, including *A. tortilis*, *A. salicina*, *A. hamulosa*, and *A. laeta* collected from eastern Saudi Arabia (about 700–1000 km from Riyadh), revealed that *A. laeta*, *A. tortilis*, and *A. hamulosa* exhibit cytotoxic effects against breast cancer and HepG2 liver cancer cell lines, as well as antimicrobial activity against *Escherichia coli*, *Staphylococcus aureus*, *Pseudomonas aeruginosa*, and *Candida albicans* (Alajmi et al. 2017). Other investigations have shown allelopathic effects of *Acacia* flower extracts on *Hordeum murinum* (Abd-ElGawad and El-Amier 2015). In Egypt, aqueous flower extracts of *A. saligna* exhibited limited antioxidant and antibacterial actions but notable antifungal potential (Al-Huqail et al. 2019). Additionally, seeds of the Chinese species *A. confusa* were reported to contain a protein with pronounced antifungal potential against *Rhizoctonia solani* (Sze Kwan and Tzi Bun 2010). Extracts from *A. ehrenbergiana* have also shown marked antibacterial effects: acetone and ethanol extracts achieved up to 85% of the control activity against Gram-negative *E. coli*, acetone extract displayed 65% activity against *Bacillus* spp., and the butanol extract showed the highest activity against *S. aureus* at 65% (Thotathil et al. 2022).

Chitosan is widely recognized as a biocompatible and non-toxic polymer, making it a promising material for diverse biomedical utilizations, particularly in the design of nanoscale drug delivery systems. Chitosan nanoparticles (CS-NPs) have emerged as an effective platform to improve the biological efficacy of both natural products and conventional antibiotics (Al-Rajhi and Abdelghany 2023a). Chitosan has made notable advances as a support and wall material in nanostructured systems due to its biocompatibility, biodegradability, non-toxicity, and ability to build stable interfaces in a variety of material shapes, including nanoparticles, films, nanogels, and porous scaffolds. It has been successfully used in medicine delivery, food preservation, wound healing, and antimicrobial coatings, allowing for efficient encapsulation, protection, and controlled release of biologically active substances (Castro-Muñoz and Cabezas 2026). Compared to other biopolymers, chitosan is an ideal wall material for improving the stability, bioavailability, and functionality of encapsulated product due to its cationic amino groups, strong mucoadhesive behavior, inherent antimicrobial activity, and high tunability via chemical and green processing strategies (Eranda et al. 2024). Intermolecular interactions among its polymeric matrix and immobilized bioactive components—such as electrostatic attractions, hydrogen bonding, and physical entrapment—strongly control chitosan's efficacy as a wall and support material. These interactions improve adhesion, stability, and long-term release of plant-derived compounds, as extensively shown in gelatin–chitosan composite systems employed for edible films and coatings in food biopreservation uses (Eranda et al. 2024).

Numerous studies have demonstrated this synergistic effect, where incorporation of plant-derived constituents into CS-NPs significantly improved their functional properties. Examples include extracts of marjoram, savory, and oregano (Negi and Kesari 2022), sage (Silva et al. 2015), *Leucas aspera* (Devi et al. 2015), *Aloe vera* gel (Yahya et al. 2022). These findings highlight the consistent enhancement of biological functions when natural extracts are combined with CS-NPs, supporting their potential as versatile carriers in both medicinal and food-related applications.

The human stomach is colonized with *Helicobacter pylori*, which is closely linked to a number of gastrointestinal illnesses. According to reports, *H. pylori* colonize almost half of the world's population, with prevalence rates being highest in underdeveloped nations (Dunn et al. 1997; Bakri et al. 2024; Alsalamah et al. 2025b; Al-Rajhi et al. 2023a). Remarkably, only about 15% of infected people develop symptoms such as peptic ulcers, gastritis, and in extreme situations, stomach cancer (Bhandari and Crowe 2012). The global health burden posed by cancer and *H. pylori*-associated diseases necessitates the search for novel, effective, and safe therapeutic agents. Thus, there is a pressing need to develop advanced formulations that can improve the stability and bioactivity of *A. seyal* seed extract, while systematically evaluating its potential as an anticancer, anti-*H. pylori*, and antioxidant agent. This study proposes a basic and translational viewpoint on the application of chitosan-based nanocarriers to improve the biological activity of bioactive chemicals obtained from plants. The paper presents chitosan nanoparticles loaded with *A. seyal* seed extract as a proof-of-concept nanoformulation, showing how biopolymer-assisted encapsulation can greatly enhance antioxidant, anti-*H. pylori*, and antineoplastic properties. Molecular docking studies against *H. pylori* urease were carried out to determine probable binding mechanisms and molecular interactions of the bioactive ingredients, providing mechanistic insight into the observed anti-*H. pylori* efficacy.

## Material and methods

### Collection and identification of *Acacia seyal* seeds

The mature seeds of *Acacia seyal* were collected from Saudi Arabia. The collected seeds of the plant were taxonomically identified and authenticated by a botanist M. REMESH at Jazan university, Saudi Arabia. The seeds were thoroughly cleaned, air-dried at room temperature, and ground into a fine powder for extraction. The powdered seeds (10 gm) were subjected to extraction using Soxhlet extraction with 100 mL of ethanol as the solvent for 60 min. The obtained extract [(17.20 ± 0.08 (% *w*/*w*)] was filtered and concentrated under reduced pressure using a rotary evaporator, then stored at 4°C until further use.

### HPLC analysis of flavonoids and polyphenols in *A. seyal* seed extract

The flavonoid and polyphenolic constituents of *A. seyal* seed extract were analyzed and quantified using high-performance liquid chromatography (HPLC). The analysis was conducted on an Agilent 1260 Infinity II HPLC system (USA) equipped with a diode array detector, with detection carried out at 280 nm, which is appropriate for phenolic compound monitoring. Separation was achieved using a reversed-phase C18 column (4.6 × 250 mm, 5 µm particle size) maintained at 40 °C. The injection volume for each run was 10 µL. The mobile phase consisted of 0.05% trifluoroacetic acid in ultrapure water (Solvent A) and methanol (Solvent B). Elution was performed using a linear gradient program as follows: 0–5 min, 85% A; 5–8 min, 65% A; 8–12 min, 65% A; 12–15 min, 80% A; 15–18 min, 78% A; and 18–25 min, 80% A, at a constant flow rate of 1.0 mL min⁻^1^. Identification of phenolic and flavonoid compounds was based on comparison of retention times and UV–visible spectral characteristics with those of authentic reference standards, including gallic acid, catechin, and quercetin. Quantitative determination was performed using external standard calibration curves prepared from known concentrations of the corresponding reference compounds (Yahya et al. 2022).

### Preparation of extract-loaded nanoparticles (ASSE@CS-NPs)

For loading *A. seyal* seed extract (ASSE), the extract (dissolved in ethanol: water, 70:30) was added to the chitosan solution (1.0 mg/ml) (sourced from Primex, Siglufjordur, Iceland) before the addition of Sodium tripolyphosphate (TPP). The TPP solution (1.0 mg/mL) was then added dropwise under vigorous stirring (pH 4.7), leading to the encapsulation of the extract within the chitosan nanoparticles. The obtained ASSE-loaded CS-NPs were centrifuged, washed to remove unbound extract, and freeze-dried.

### Fourier-transform infrared (FTIR) analysis

The functional groups of ASSE, CS-NPs, and ASSE@CS-NPs were analyzed using FTIR. Samples were lyophilized and finely ground prior to measurement. Spectra were recorded with an ATR-FTIR spectrophotometer equipped with a diamond crystal, operating in the range of 4000–400 cm⁻^1^ at a resolution of 4 cm⁻^1^ with 32 scans per sample.

### Anti-*Helicobacter pylori* activity by agar well diffusion method

The anti-*H. pylori* activity of ASSE, CS-NPs, and the ASSE@CS-NPs was evaluated by the agar well diffusion assay. A reference strain of *H. pylori* (ATCC 43504) was cultured on Mueller Hinton agar plates supplemented 10% sheep blood and 1% IsoVitalex under microaerophilic conditions (10% CO₂) at 37 °C for 48–72 h. Fresh colonies were suspended in sterile phosphate-buffered saline and adjusted to 0.5 McFarland standard (1 × 10^8^ CFU/mL). The bacterial suspension was streak uniformly over the agar surface, and 6-mm wells were aseptically punched into the medium with a sterile cork borer. Each well was filled with 100 μL of the test samples. Sterile 1% DMSO served as the negative control, clarithromycin (10 μg/mL) as the positive control, and blank CS-NPs as the polymer control. Plates were kept at 4 °C for 30 min to facilitate pre-diffusion of the samples into the agar and were then incubated at 37 °C under microaerophilic conditions for 48–72 h. Following incubation, the diameters of the appeared inhibition zones were measured in millimeters (Yahya et al. 2022). The minimum inhibitory concentration (MIC) and minimum bactericidal concentration (MBC) of ASSE, CS-NPs, and ASSE@CS-NPs were detected by broth microdilution assay. *H. pylori* colonies were suspended in Brucella broth supplemented with 10% fetal bovine serum and adjusted to 0.5 McFarland standard (1 × 10^8^ CFU/mL). Two-fold serial dilutions of the test samples (0.031–8 mg/mL extract equivalents) were prepared in sterile 96-well microtiter plates, and each well was inoculated with an equal volume of *H. pylori* cells suspension (5 × 10^5^ CFU/mL). Medium without bacterial inoculum in well served as sterility controls, wells with bacteria and medium only served as growth controls, and wells with clarithromycin were included as positive controls. Plates were incubated at 37 °C under microaerophilic conditions for 72 h, and bacterial growth was visually assessed by turbidity and confirmed by measuring absorbance at 600 nm. The MIC was defined as the lowest concentration of the sample at which no visible growth was observed compared with the growth control. To determine the MBC, aliquots of 10 μL from wells showing no visible growth were sub-cultured onto on Mueller Hinton agar plates supplemented 10% sheep blood and incubated under the same conditions for 72 h. The MBC was defined as the lowest dose at which no bacterial colonies were recovered, corresponding to ≥ 99.9% reduction in the initial inoculum.

### Antioxidant activity by DPPH assay & ABTS free radical scavenging test

The antioxidant potential of ASSE, CS-NPs, and ASSE@CS-NPs was evaluated using the 2,2-diphenyl-1-picrylhydrazyl (DPPH) radical scavenging method. A 0.1 mM solution of DPPH was freshly prepared in methanol. Different doses of the test samples (ranging from 1.95 to 1000 μg/mL) were prepared in methanol, and 1 mL of each was mixed with 1 mL of the DPPH solution in sterile tubes. The mixtures were vortexed briefly and incubated in the dark at 25 °C for 30 min. The absorbance was measured at 517 nm against methanol as the blank using a UV–Vis spectrophotometer (Abdel Ghany and Hakamy 2014). Ascorbic acid was used as the reference antioxidant, and methanol alone served as the control. The percentage inhibition of DPPH radicals was calculated using the formula:

1 $${\% Scavenging Activity}=\frac{\mathrm{Acontrol}-\mathrm{Asample}}{\mathrm{Acontrol}}\times 100$$

where Acontrol: is the absorbance of the DPPH solution without sample, and Asample: is the absorbance with the test sample. The half maximal inhibitory concentration (IC_50_) was determined from the concentration–response curve by plotting percentage scavenging activity against sample concentration.

Using the procedure outlined by Hussen and Endalew (2023) the antioxidant potential of the investigated materials against ABTS was ascertained. By oxidizing ABTS with potassium persulfate, radical ABTS· + was created. A 1:1; v/v combination of potassium persulfate (4.97 mM) and ABTS (6.9 mM) was made and allowed to sit at room temperature in the dark for 17.0 h. After then, methanol was added to the mixture until its absorbance at 734 nm ranged from 1 to 1.5. 3.9 mL of the ABTS· + dilution was applied to aliquots of 0.1 mL of each sample (at Six distinct concentrations: 50–30 μg/ml; two duplicates per sample and concentration). A UV-30 spectrophotometer was used to quantify the absorbance drop at 734 nm. ABTS· + was used to prepare the blank. The findings were reported as milligrams of quercetin equivalents per milligram of dry weight. Each solvent extract's reactivity at different concentrations was contrasted with ascorbic acid. At least three attempts were made at each measurement. The following formula was used to determine the percentage scavenging of ABTS + radical for various extract and standard concentrations (50 to 300 μg/mL). The percentage inhibition of ABST radicals was calculated using the formula:

2 $${\% Scavenging Activity}=\frac{\mathrm{Acontrol}-\mathrm{Asample}}{\mathrm{Acontrol}}\times 100$$

where Asample is the absorbance of the mixture of sample/standard and ABTS, and Acontrol is the absorbance of a mixture of 10 mL of (7 mM ABTS, 2.45 mM K2S2O8) with blank solvents. IC_50_ was used to express the sample's antioxidant action against ABTS +·.

### Cytotoxicity of ASSE, CS-NPs, and ASSE@CS-NPs (MTT assay)

The antineoplastic activity of ASSE, CS-NPs, and ASSE@CS-NPs was evaluated against human colorectal carcinoma cells (HCT116) and WI38 cell line (normal human fetal lung fibroblasts) (obtained from the American Type Culture Collection (ATCC, Manassas, VA, USA) using the MTT colorimetric assay. HCT116 cells were maintained in RPMI medium supplemented with 2% heat-inactivated fetal bovine serum, 1% penicillin–streptomycin, and 2 mM L-glutamine at 37 °C in a humidified incubator with 5% CO₂. Cells in the exponential growth phase were harvested with trypsin–EDTA, counted, and seeded into sterile 96-well plates at 5 × 10^3^ cells/well in 100 µL complete medium; plates were incubated for 24 h to allow attachment. Stock solutions of ASSE were prepared in sterile DMSO and diluted with medium to the required concentrations, keeping the final DMSO ≤ 0.5% v/v. CS-NPs and ASSE@CS-NPs were dispersed in sterile PBS, adjusted to near-neutral pH (7.0), and filtered (0.22 µm). Cells were then treated (in quadruplicate wells) with 100 µL of each test preparation over a two-fold serial dilution range (7.81–1000 µg/mL) of tested samples. Vehicle control (medium with 0.5% DMSO), polymer control (blank CS-NPs at the highest chitosan level), and a reference drug control (doxorubicin, 0.1–10 µM) were included on every plate. After 24 h exposure, 10 µL of MTT reagent (5 mg/mL in PBS) was added per well and plates were incubated for 3–4 h at 37 °C until purple formazan crystals were evident. The medium was carefully removed, and 100 µL DMSO was added to dissolve the formazan with gentle shaking for 10 min. Absorbance was read at 570 nm (Abdelghany et al. 2019). Morphology was examined by phase-contrast microscopy to verify cytopathic effects of samples. The cytotoxicity % was calculated from the next equation:

3 $$\text{Cytotoxicity }(\mathrm{\%}) =100-\frac{\text{A sample}}{\text{A control}}\times 100$$

### Molecular docking analysis

The crystal structure of *H. pylori* urease (PDB ID: 6ZJA; resolution 2.0 Å) was retrieved from the RCSB Protein Data Bank (https://www.rcsb.org). Protein preparation was carried out using Molecular Operating Environment (MOE) version 2019.01 (Chemical Computing Group, Montreal, Canada), including removal of water molecules, protonation at physiological pH, and energy minimization. Finally, the site finder created the active binding sites, which served as the binding pocket's dummy sites. Ligands namely methyl gallate (PubChem CID: 7428) and a chitosan (PubChem CID: 129,662,530) were prepared using MOE’s builder tool, energy minimized, and saved in mdb format. Molecular docking was conducted using the Triangle Matcher placement method and the stiff receptor atoms were docked for 100 ns. The GBVI/WSA dG processes were used for rescoring, with the London dG acting as a scoring function. For each ligand–protein pair, several postures were created, and the top five were chosen for further investigation. 2D and 3D interaction diagrams were created to show how both ligands attach to each protein's active regions. These graphical representations focused on specific interactions. The docked complexes were examined to identify the interactions between the studied ligands and the active site residues of the protein.

### Statistical analysis

All experiments were carried out in triplicate, and the data were expressed as mean ± standard deviation (SD). Statistical analysis was performed using one-way analysis of variance (ANOVA) to determine significant differences among treatments.

## Results and discussion

### Phytochemical characterization

**Fig. 1:**
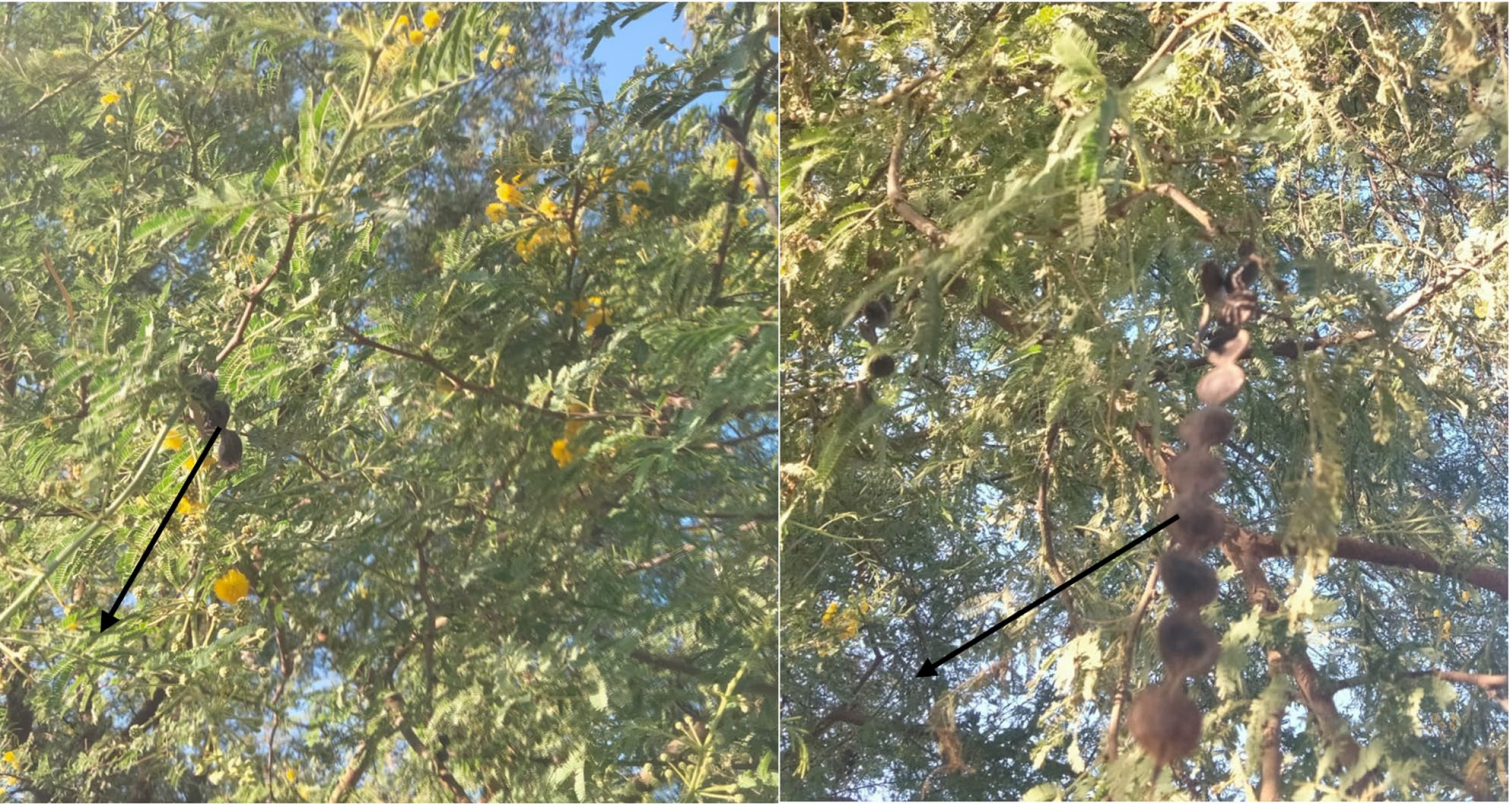
*Acacia seyal* tree with seeds (black arrow)

**Fig. 2:**
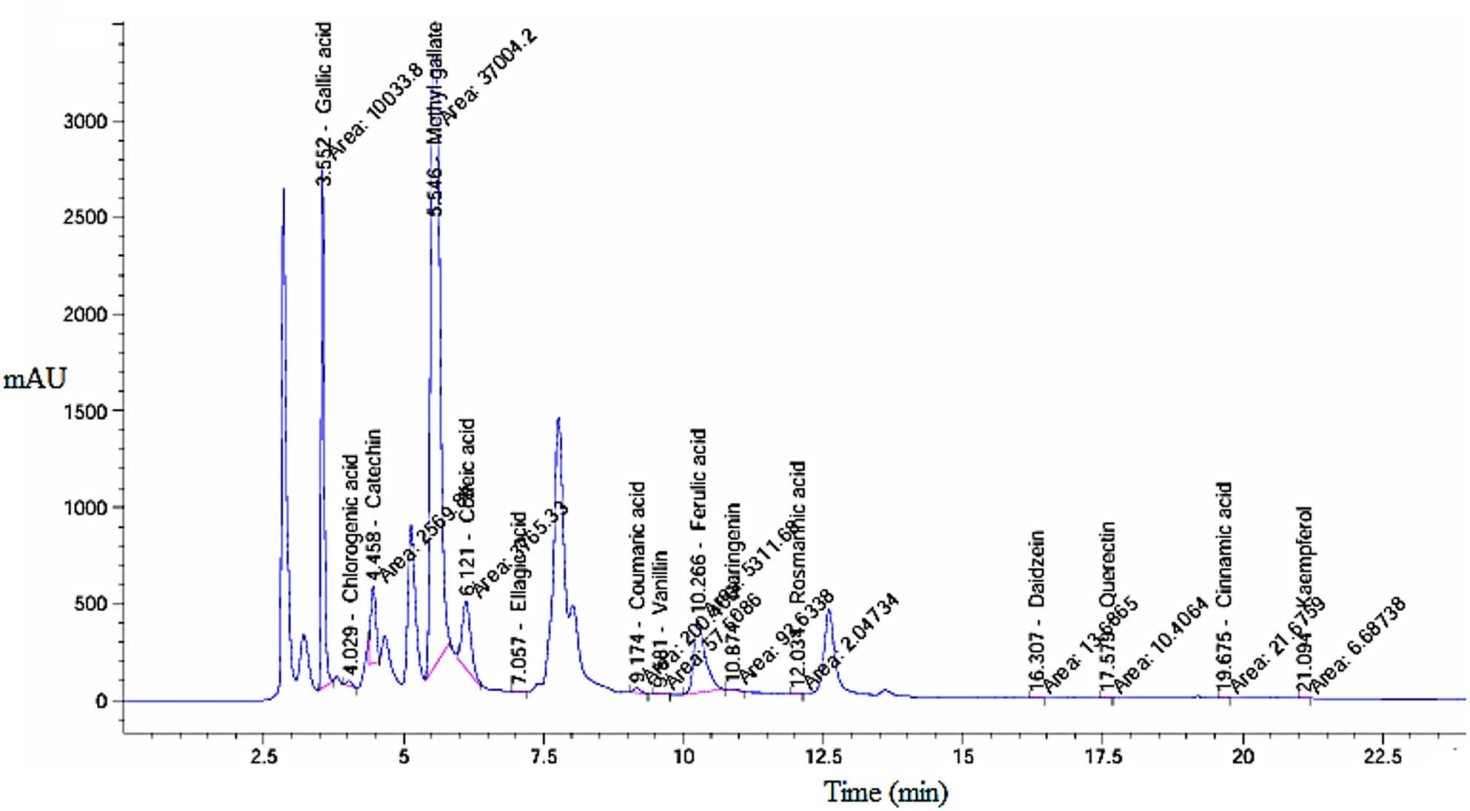
Chromatographic profile of ASSE by HPLC

The phytochemical analysis of ASSE (Fig. 1) revealed the presence of a diverse range of phenolic acids and flavonoids, with considerable variation in their relative abundance (Table 1 and Fig. 2). Among the identified compounds, methyl gallate was predominant, representing 62.40% of the total chromatographic area and achieving the highest concentration (20.98 mg/g). This is followed by gallic acid (16.92%, 7.27 mg/g), catechin (4.33%, 5.97 mg/g), and caffeic acid (6.35%, 2.25 mg/g). These results suggest that hydrolyzable tannin derivatives and simple phenolic acids constitute the major fraction of the extract. Other compounds such as ferulic acid (8.95%, 3.07 mg/g), rosmarinic acid (3.45%, 0.003 mg/g), and trace flavonoids like quercetin and kaempferol were detected in much smaller quantities. Although present at low levels, these flavonoids may act synergistically with major phenolics to enhance biological activity, as previously reported in studies of multi-compound plant extracts (Thotathil et al. 2022; Al-Rajhi et al. 2023a; Hussen and Endalew 2023). Several phenolics commonly reported in green tea, including syringic acid, rutin, and hesperetin, were not detected in this analysis. Their absence could be attributed to the extraction parameters of the used method. Additionally, environmental factors, plant maturity, and post-harvest handling may influence the phytochemical profile. The retention times of detected compounds ranged from 3.55 min for gallic acid to 21.10 min for kaempferol, reflecting the chemical diversity and polarity differences among the compounds. Such variation also supports the conclusion that the extract contains a broad spectrum of phenolics, each potentially contributing distinct bioactivities. Analysis of *Acacia nilotica* ripe fruits revealed that methyl ester of gallic acid was the predominant compounds, with catechin-7-gallate and catechin also being abundant (Foyzun et al. 2022). Likewise, Kaur et al. (Kaur et al. 2022) employed HPLC to characterize the bark extract of *A. nilotica*, identifying a wide spectrum of phenolic compounds, including myricetin, ferulic acid, rutin, kaempferol, gallic acid, quercetin, betulin, epicatechin, and catechin. A wide range of phytochemicals, including saponins, proteins, phenolic compounds, anthocyanins, and flavonoids, have been reported in appreciable amounts in the pods, flowers, and leaves of *A. seyal* (Magnini et al. 2020). The foliage of this species is particularly rich in phenolic acids such as salicylic, gallic, caffeic, *p*-coumaric, ferulic and 3,4-dihydroxybenzoic acids (Magnini et al. 2020). Additionally, investigations on the stem bark of the Djibouti type of *A. seyal* revealed the presence of epicatechin, catechin, lupeol, stigmasterol, campesterol, oleamide, and clionasterol (Elmi et al. 2020).

**Table 1:** HPLC-detected phytochemical compounds in ASSE with their area percentage, retention time, and concentration

Detected compound	Area (%)	Retention time (min)	Concentration (mg/g)
Gallic acid	16.92	3.55	7.27
Chlorogenic acid	0.29	4.02	0.24
Catechin	4.34	4.46	6.0
Methyl gallate	62.40	5.55	21.0
Caffeic acid	6.35	6.12	2.25
Ellagic acid	0.07	7.06	0.04
Coumaric acid	0.34	9.17	0.07
Vanillin	0.10	9.58	0.02
Ferulic acid	8.96	10.27	3.07
Naringenin	0.16	10.88	0.09
Rosmarinic acid	3.45	12.03	0.003
Daidzein	0.02	16.30	0.001
Querectin	0.02	17.58	0.010
Cinnamic acid	0.04	19.68	0.002
Kaempferol	0.01	21.10	0.001

### FTIR analysis

**Fig. 3:**
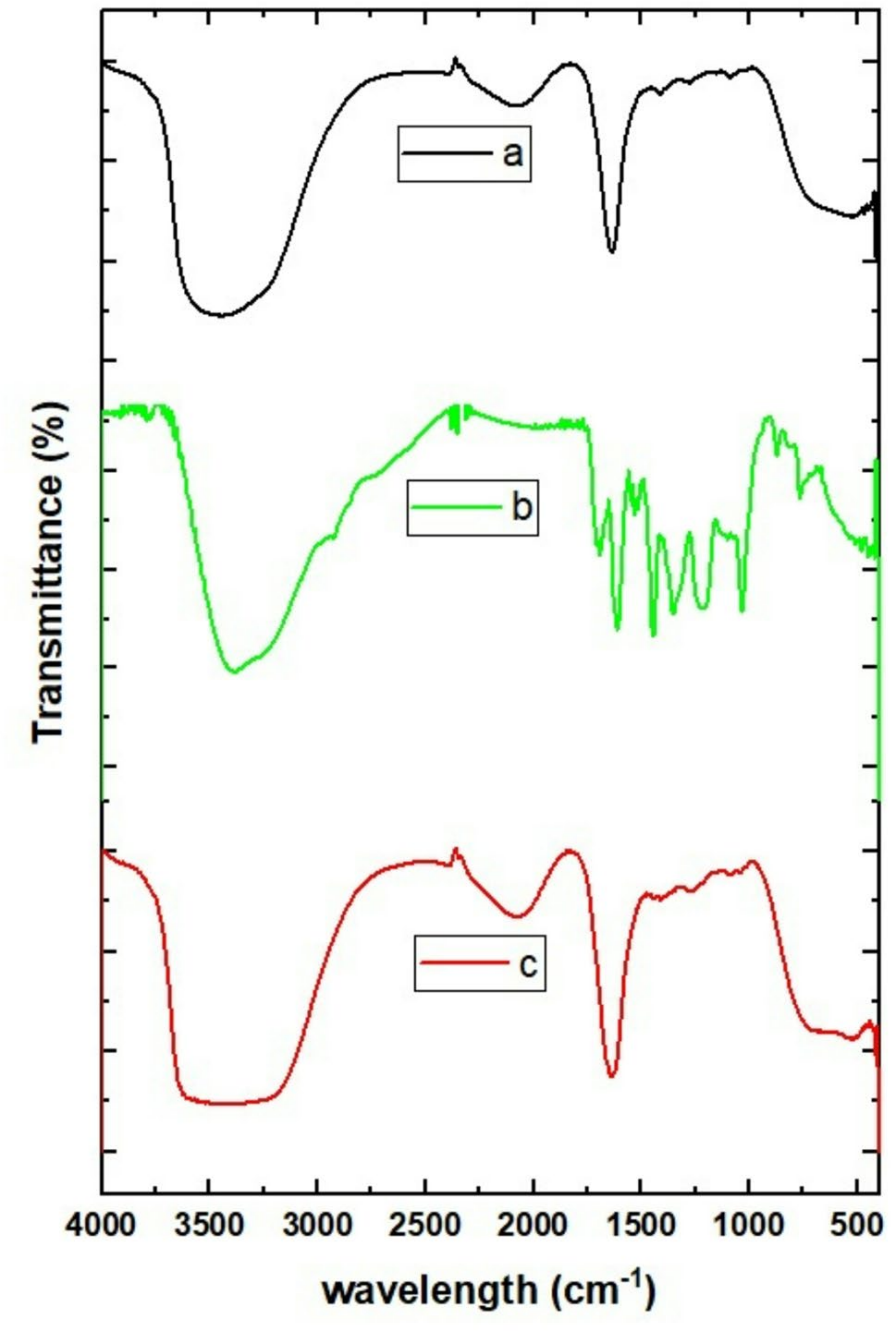
FTIR analysis of CS-NPs (**a**), ASSE (**b**), and ASSE@CS-NPs (**c**) confirming characteristic functional groups

The FTIR spectrum (Fig. 3) of the ASSE revealed characteristic absorption bands associated with its phytochemical constituents. A broad peak around 3360 cm⁻^1^ corresponds to O–H stretching vibrations of hydroxyl groups from phenolics and flavonoids. The bands at 2920–2850 cm⁻^1^ indicate C–H stretching of aliphatic groups. A sharp peak at approximately 1635 cm⁻^1^ corresponds to C = O stretching of carbonyl groups, while the bands at 1600–1500 cm⁻^1^ confirm the presence of aromatic C = C stretching vibrations. Furthermore, absorption around 1070–1040 cm⁻^1^ is attributed to C–O stretching of alcohols, esters, and glycosidic linkages. The FTIR spectrum of CS-NPs displayed a broad absorption band near 3350 cm⁻^1^, assigned to overlapping O–H and N–H stretching vibrations, indicating strong hydrogen bonding. Peaks observed at 2920 cm⁻^1^ represent C–H stretching vibrations. The band near 1640 cm⁻^1^ is attributed to the amide I group (C = O stretching), while the 1550 cm⁻^1^ band corresponds to amide II (N–H bending). The strong peak observed around 1075–1030 cm⁻^1^ is due to C–O–C stretching vibrations of the glycosidic bonds in the chitosan backbone. The characteristic peaks of CS-NPs in our study were matching consistently with previous study (Chang et al. 2021). For the ASSE@CS-NPs, the FTIR spectrum demonstrated the occurrence of combined features from both CS-NPs and the ASSE, with noticeable peak shifts and intensity variations. The broad band at 3360 cm⁻^1^ became more intense and slightly shifted, suggesting hydrogen bonding interactions among the hydroxyl groups of the extract and the amino/hydroxyl groups of CS-NPs. The characteristic bands at 1635 cm⁻^1^ (C = O stretching) and 1550 cm⁻^1^ (N–H bending) persisted but showed reduced intensity, demonstrating encapsulation of the ASSE within the polymeric matrix. The aromatic C = C stretching around 1600 cm⁻^1^ and improved C–O stretching among 1070–1040 cm⁻^1^ further established the successful incorporation of the phytochemicals into the CS-NPs. Overall, the observed shifts and peak overlaps confirm effective encapsulation of ASSE within CS-NPs through hydrogen bonding and electrostatic interactions. The present results are consistent with previous reports (Chang et al. 2021; Surendhiran et al. 2020), which also demonstrated the successful entrapment of plant-derived bioactive compounds within chitosan-based nanoparticles.

### Anti-*H. pylori* activity of CS-NPs, ASSE, and ASSE@CS-NPs

**Fig. 4:**
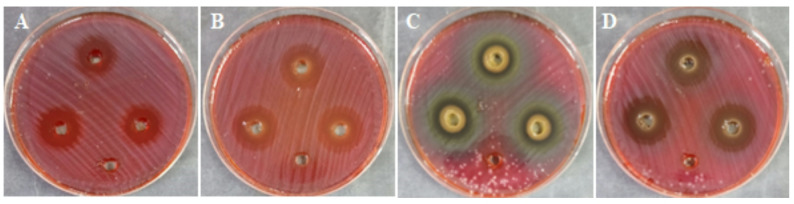
Comparative anti-*H. pylori* activity of CS-NPs, ASSE, and ASSE@CS-NPs assessed by the well diffusion method (**A**: Control, **B**: CS-NPs, **C**: ASSE, **D**: ASSE@CS-NPs). Well without inhibition zone in each plate represent negative control

The antibacterial activity of CS-NPs, ASSE, and ASSE@CS-NPs against *H. pylori* was documented in Table 2 and Fig. 4. All treatments demonstrated measurable inhibition zones, with the combination of ASSE@CS-NPs exhibiting the largest zone of inhibition (23.7 ± 0.6 mm), followed by chitosan nanoparticles alone (20.7 ± 0.6 mm), the extract alone (19.7 ± 0.6 mm), and the control (18.7 ± 0.6 mm). MIC values revealed that the ASSE@CS-NPs treatment required the lowest concentration (15.62 μg/mL) to inhibit *H. pylori* growth, which was two-fold lower than that of CS-NPs or ASSE alone (31.25 μg/mL). Notably, the combination also achieved MBC of 15.62 μg/mL, yielding an MBC/MIC index of 1, indicating a strong bactericidal effect. The superior performance of the chitosan–extract formulation can be attributed to a synergistic effect between the cationic nature of chitosan nanoparticles, which enhances bacterial cell membrane permeability, and the potent phenolic compounds in ASSE, notably methyl gallate, gallic acid, and catechin. This combination likely facilitates greater penetration and interaction of bioactive molecules with intracellular targets, thereby accelerating bacterial cell death. These findings are consistent with previous studies reporting that polyphenol-loaded nanoparticles display enhanced antimicrobial efficacy due to improved stability, sustained release, and targeted delivery of active compounds. The marked reduction in both MIC and MBC values for the combination treatment underscores its potential as a promising alternative or adjunct therapy against *H. pylori*, particularly in the face of increasing antibiotic resistance.

**Table 2:** Anti-*H. pylori* activity of CS-NPs, ASSE, and ASSE@CS-NPs with MIC and MFC values (mean ± SD). Different small letters above numbers in the same columns refer to significant difference where *P* ≤ 0.05

Treatment	Zone of inhibition (mm)	MIC (μg/mL)	MBC (μg/mL)	MBC/MIC Index
CS-NPs	20.7 ± 0.6^a^	31.25^a^	62.5^a^	2
ASSE	19.7 ± 0.6^a^	31.25^a^	62.5^a^	2
ASSE@CS-NPs	23.7 ± 0.6^b^	15.62^b^	15.62^c^	1
Control (clarithromycin)	18.7 ± 0.6^c^	15.62^b^	31.25^b^	2

Previous studies have reported limited antibacterial activity of aqueous *A. seyal* extracts and moderate activity of methanolic extracts, with stronger effects observed mainly against *Staphylococcus aureus* and *Corynebacterium urealyticum* (Elmi et al. 2020). In agreement with these findings, our results demonstrate that incorporation of *A. seyal* seed extract into chitosan nanoparticles significantly enhanced its antibacterial efficacy, supporting the role of CS-NPs in improving the bioactivity of *Acacia*-derived compounds. Mashraqi (Mashraqi 2023) demonstrated that encapsulating *Anethum graveolens* extract within CS-NPs enhanced its antibacterial performance against several food-borne pathogens. Similarly, Qanash et al. (Qanash et al. 2023a) highlighted that *Artemisia judaica* extract, when incorporated into CS-NPs, exhibited greater antimicrobial potential against human pathogens compared to the crude extract alone. In another investigation, Yahya et al. (Yahya et al. 2022) found that loading *Aloe vera* gel into CS-NPs markedly suppressed *H. pylori* growth, whereas the untreated extract had a weaker effect. These findings collectively indicate that the superior biological activity may be linked to the nanoscale features of CS-NPs—particularly their small particle size and high surface charge—which facilitate closer interaction between the bioactive compounds and microbial cell walls.

### Antioxidant effect of CS-NPs, ASSE, and ASSE@CS-NPs via DPPH scavenging and ABTS examination

DPPH radical scavenging activities (Table 3) and ABTS levels (Table 4) of CS-NPs, ASSE, and ASSE@CS-NPs, compared to the standard antioxidant ascorbic acid, across a concentration range of 1.95–1000 µg/mL for DPPH and 50–300 µg/mL for ABTS were recorded. A clear dose-dependent increase in antioxidant activity for all tested samples was recorded. DPPH outcomes revealed that at the lowest tested concentration (1.95 µg/mL), the ASSE extract and the ASSE@CS-NPs already exhibited high scavenging activities (39% and 43%, respectively), approaching the ascorbic acid activity (43%). In contrast, chitosan nanoparticles alone displayed minimal activity (6%), indicating that chitosan nanoparticles have limited intrinsic radical scavenging capacity at low doses. As the dose increased, ASSE@CS-NPs maintained strong antioxidant activity, with ASSE@CS-NPs consistently showing slightly higher or comparable percentages to the plant extract alone, particularly in the low-to-mid concentration range (1.95–31.25 µg/mL). At higher concentrations (≥ 250 µg/mL), both ASSE and ASSE@CS-NPs achieved scavenging percentages above 80%, comparable to ascorbic acid. Notably, at 1000 µg/mL, the ASSE@CS-NPs achieved 96.1% scavenging, almost identical to the standard (98%), while chitosan nanoparticles alone remained significantly lower (74%). ABTS results reveled that ascorbic acid IC_50_ was at 13 ± 0.2 µg/mL, ASSE IC_50_ = 18 ± 1 µg/mL, CS-NPs IC_50_ = 37 ± 0.2 µg/mL, and ASSE@CS-NPs IC_50_ was at 15 ± 0.1 µg/mL. The calculated IC_50_ values further confirm the potency differences where ascorbic acid, ASSE, CS-NPs, and ASSE@CS-NPs were 3, 5, 74, and 3 µg/mL, respectively. The significantly lower IC_50_ of the ASSE@CS-NPs compared to the ASSE alone suggests that encapsulation in chitosan nanoparticles enhanced the antioxidant efficacy, possibly through improved bioavailability and stabilization of active compounds. The antioxidant potential of different parts of *A. seyal* has been reported in the literature (Al-Rajhi et al. 2023b; Subhaswaraj et al. 2017). Previous reports indicated that methyl gallate as a major content in ASSE exhibits antimicrobial, antioxidant, and anti-inflammatory effects (Correa et al. 2020; Ahmed et al. 2021). Previous reports have suggested that the encapsulation of plant extracts within CS-NPs not only enhances their antioxidant potential but also improves their stability, ensuring a more sustained biological effect (Negi and Kesari 2022).

**Table 3:** Comparative DPPH radical scavenging effect of CS-NPs, ASSE, and ASSE@CS-NPs across different concentrations and standard compounds

Concentration (µg/mL)	Antioxidant via DPPH scavenging%			
	Ascorbic Acid	ASSE	CS-NPs	ASSE@CS-NPs
0.0	0 ± 0.0^a^	0 ± 0.0^a^	0 ± 0.0^a^	0 ± 0.0^a^
1.95	43 ± 2.0^a^	39 ± 0.2^a^	6 ± 0.2^b^	43 ± 1^a^
3.9	52 ± 2.0^a^	47 ± 1^a^	13 ± 1^b^	51 ± 1^a^
7.81	58 ± 1.3^a^	55 ± 2^a^	21 ± 0.2^b^	56 ± 2^a^
15.62	66 ± 2.7^a^	62 ± 1^a^	29 ± 0.3^b^	60 ± 0.2^a^
31.25	73 ± 2.0^a^	68 ± 1^a^	34 ± 1^b^	67 ± 2^a^
62.5	80 ± 1.0^a^	69 ± 0.3^a^	39 ± 2^a^	72 ± 1^a^
125	87 ± 0.4^a^	77 ± 2^a^	49 ± 1^a^	76 ± 2^a^
250	94 ± 1.8^a^	84 ± 3^a^	57 ± 0.1^a^	83 ± 3^a^
500	98 ± 2.2^a^	91 ± 1^a^	65 ± 2^a^	92 ± 2^a^
1000	98 ± 0.4^a^	93 ± 2^a^	74 ± 2^a^	96 ± 0.3^a^
IC_50_ µg/mL	3 ± 0.3	5 ± 0.4	74 ± 2	3 ± 0.3

Different small letters above numbers in the same raw refers to significant difference where *P* ≤ 0.05

**Table 4:** Comparative ABTS radical scavenging impact of CS-NPs, ASSE, and ASSE@CS-NPs across various levels and standard

Concentration (µg/mL)	Antioxidant via ABTS scavenging%			
	Ascorbic Acid	ASSE	CS-NPs	ASSE@CS-NPs
50	43 ± 1^a^	40 ± 1^a^	4 ± 1^b^	41 ± 1^a^
100	54 ± 2^a^	51 ± 1^a^	14 ± 2^b^	53 ± 2^a^
150	65 ± 1^a^	61 ± 1^a^	35 ± 1^b^	63 ± 1^a^
200	72 ± 1^a^	70 ± 1^a^	42 ± 1^a^	71 ± 1^a^
250	83 ± 1^a^	82 ± 1^a^	53 ± 1^a^	81 ± 1^a^
300	91 ± 1^a^	90 ± 1^a^	61 ± 1^a^	92 ± 1^a^
IC_50_ µg/mL	13 ± 0.2	18 ± 1	37 ± 0.2	15 ± 0.1

Various small letters above numbers in the same raw refers to dramatic difference where *P* ≤ 0.05

### Cytotoxic response of HCT116 cells to CS-NPs, ASSE, and ASSE@CS-NPs treatment

**Fig. 5:**
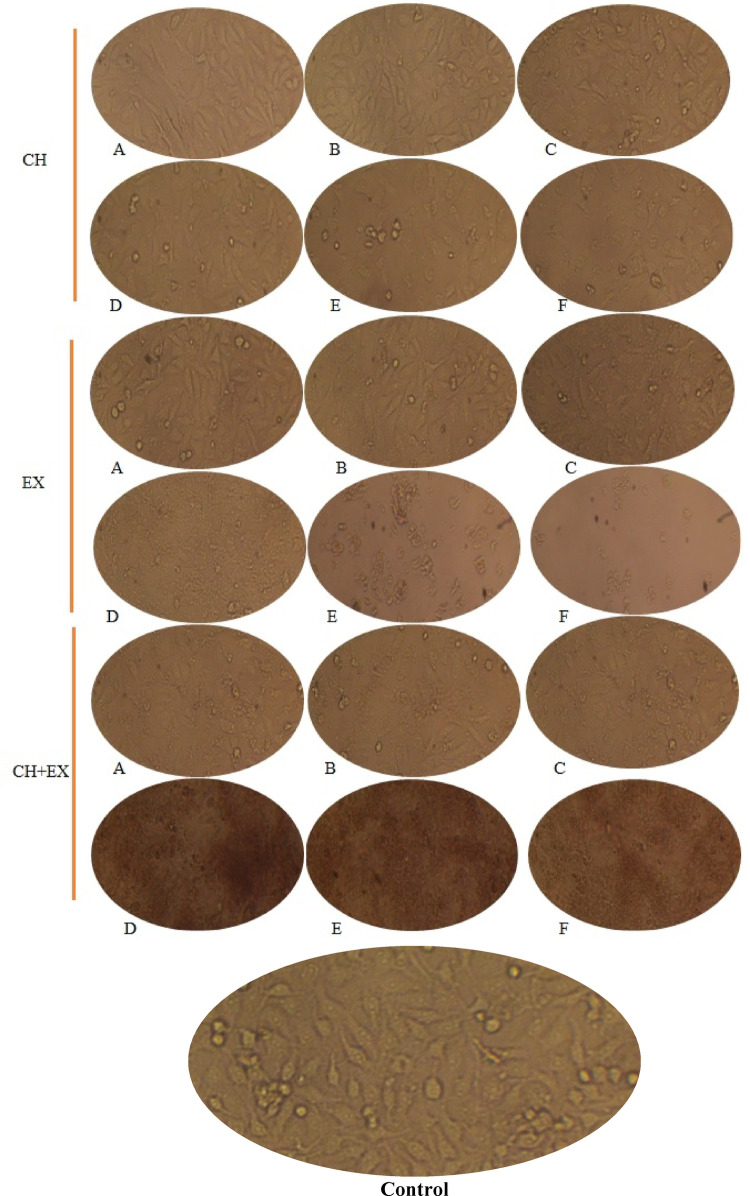
Cytotoxic response of HCT116 cells to CS-NPs (CH), ASSE (EX), and ASSE@CS-NPs (CH + EX) treatment compared to control

In vitro cytotoxicity of CS-NPs, ASSE, and ASSE@CS-NPs against the HCT116 human colorectal carcinoma cell line across a concentration range of 7.81–1000 µg/mL was recorded (Table 5). At very low concentrations (≤ 15.26 µg/mL), both chitosan nanoparticles and Acacia extract exhibited negligible cytotoxicity, with cell viability close to 100%. In contrast, the ASSE@CS-NPs formulation demonstrated a notable early effect, showing 6% inhibition at 7.81 µg/mL and a dramatic increase to 46% inhibition at 15.26 µg/mL, suggesting a strong synergistic cytotoxic potential even at sub-therapeutic doses. As concentration increased, chitosan nanoparticles alone began to show moderate activity, reaching 54% inhibition at 125 µg/mL and achieving high cytotoxicity (≥ 91%) at doses ≥ 250 µg/mL. ASSE exhibited a slower potency curve, with inhibition of only 20% at 125 µg/mL but progressively rising to 66% at 1000 µg/mL, indicating a moderate dose-dependent cytotoxic effect. The ASSE@CS-NPs consistently outperformed both individual components across all tested concentrations, reaching over 80% inhibition at just 62.50 µg/mL and maintaining 92% inhibition from 125 µg/mL onwards. This high-level activity plateau suggests that most cancer cell killing occurred before the highest concentrations were reached, reflecting potent early cytotoxic action. The IC_50_ values further underscore these potency differences including 23 ± 0.3, 120 ± 2, and 588 ± 12 µg/mL using ASSE@CS-NPs, CS-NPs, and ASSE, respectively. These results indicate that while chitosan nanoparticles have limited direct cytotoxicity, their role as a nanocarrier significantly enhances the delivery and activity of ASSE bioactivates. The marked improvement in potency for the ASSE@CS-NPs formulation suggests potential synergistic mechanisms, including increased cellular uptake, controlled release of active compounds, and improved stability of phytochemicals. The micrographs seem to show cells with varying morphology and density under different concentrations. The exposed cells to CS-NPs appeared relatively intact with elongated or spindle-like shapes. On the other hand, partial cell shrinkage with rounding, indicating potential stress or cytotoxicity of extract, in addition, more pronounced changes are visible—cell detachment, fragmentation, or darker staining, suggesting stronger morphological effects of ASSE@CS-NPs (Fig. 5). All exposed cells to treatments were compared to untreated cells (control) (Fig. 5). Such effects may be attributed to the presence of bioactive phytochemicals, particularly phenolics and tannins, which exert toxic influences on cancer cells. On the other hand, while the data are not presented in the Table 5, ASSE@CS-NPs, ASSE, and CS-NPs showed negligible inhibitory effects on the WI38 cell line (normal human fetal lung fibroblasts), as reflected by their high IC_50_ values (277, 611, and 290 μg/mL, respectively), suggesting low and limited cytotoxicity toward normal cells. The cytotoxicity of *A. seyal* bark extracts on the embryonic lung cell line MRC-5 was demonstrated by a marked reduction in cell density along with distinct morphological alterations under microscopic observation (Abdel Ghany and Hakamy 2014). In our investigation, the main detected polyphenolic compound was methyl gallate in *A. seyal* seeds extract, according to Liang et al. (Liang et al. 2022), methyl gallate is widely employed in traditional applications for alleviate numerous cancer signs.

**Table 5:** Dose–response cytotoxic effects (%) of CS-NPs, ASSE, and ASSE@CS-NPs against HCT116 colorectal carcinoma cells

Concentration (µg/mL)	CS-NPs	ASSE	ASSE@CS-NPs
0.00	0.0 ± 0.0^a^	0.0 ± 0.0^a^	0.0 ± 0.0^a^
7.81	0.0 ± 0.0^a^	0.0 ± 0.0^a^	6 ± 0.02^b^
15.26	0.0 ± 0.0^a^	0.0 ± 0.0^a^	46 ± 0.1^b^
31.25	2 ± 0.02^a^	0.1 ± 0.01^a^	62 ± 0.04^b^
62.50	9 ± 0.02^a^	0.2 ± 0.01^a^	82 ± 0.13^b^
125	54 ± 0.02^a^	20 ± 0.12^b^	92 ± 0.1^a^
250	91 ± 0.1^a^	33 ± 0.03^b^	92 ± 1.02^a^
500	97 ± 0.1^a^	51 ± 0.2^a^	92 ± 0.01^a^
1000	98 ± 0.2^a^	66 ± 0.01^a^	92 ± 0.02^a^
IC_50_ (µg/mL)	120 ± 2	588 ± 12	23 ± 0.3

Different small letters above numbers in the same raw refers to significant difference where *P* ≤ 0.05

### Docking study methyl gallate and chitosan with *H. pylori* protein

**Fig. 6:**
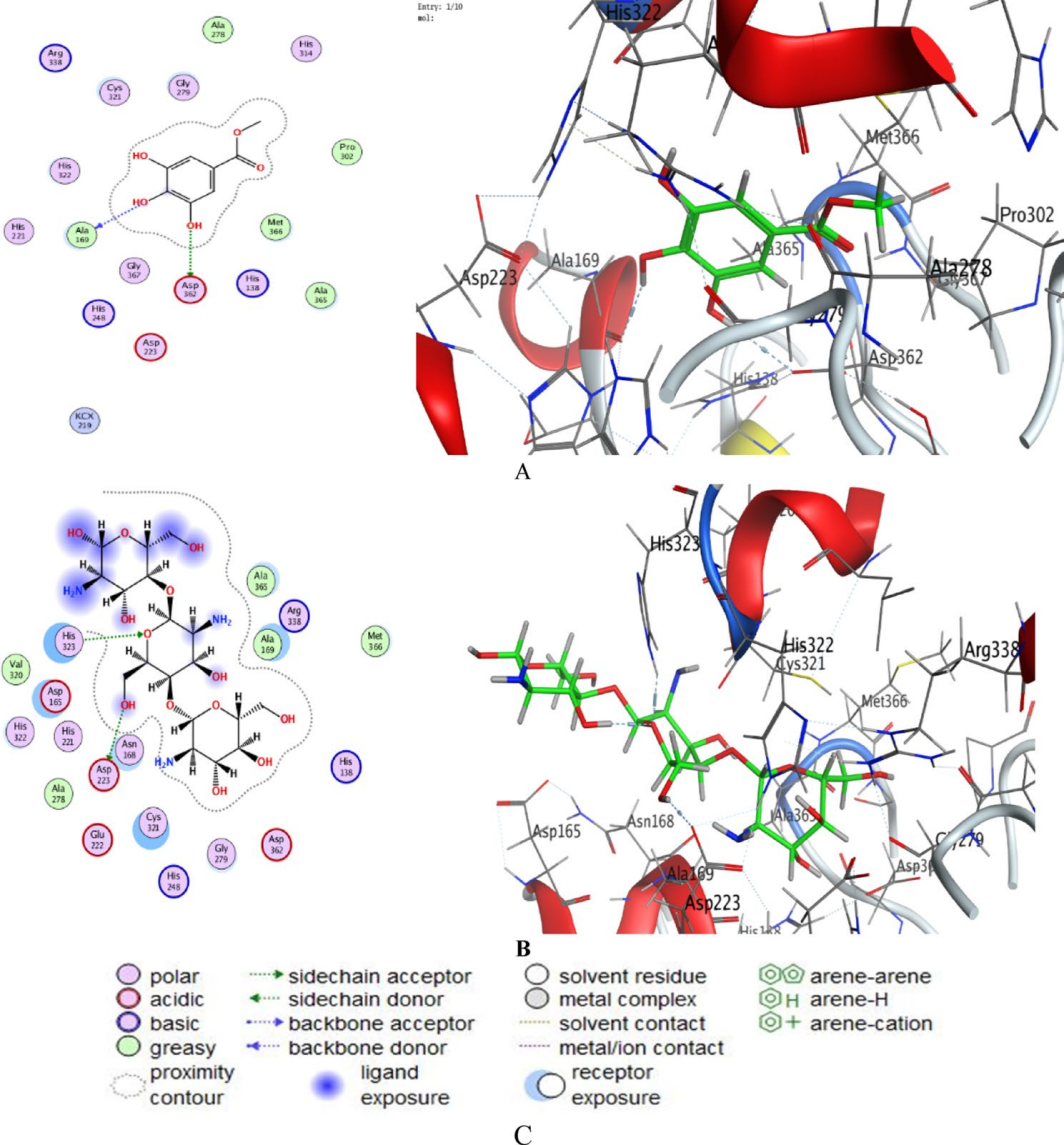
2D and 3D interaction diagrams of methyl gallate (**A**) and chitosan (**B**) with the active sites of *H. pylori* 6ZJA protein; Representative key illustrating the interaction types between ligands and selected protein receptors (**C**)

Molecular docking has emerged as a powerful computational approach for predicting the interactions between bioactive compounds and target enzymes, thereby offering valuable insights into their potential therapeutic relevance (Binsaleh et al. 2025). Urease, an essential enzyme for the survival of *H. pylori* in acidic gastric environments, represents a well-established molecular target for the design of anti-*H. pylori* agents (Qanash et al. 2023b). Previous studies have demonstrated that some phenolic compounds exert inhibitory effects (Al-Huqail et al. 2019), also chitosan and its derivatives exhibit interactions with bacterial enzymes due to their polycationic nature, enabling both antimicrobial and anti-biofilm properties (Devi et al. 2015; Mashraqi 2023; Chang et al. 2020). Against this backdrop, the current docking analysis of methyl gallate and chitosan was undertaken to explore their inhibitory potential and interaction profiles with *H. pylori* urease, thereby providing mechanistic support for their observed biological activities. Docking Scores of tested compounds were recorded in Supplementary (1), where methyl gallate exhibited docking scores ranging from − 5.25 to − 5.45 kcal/mol, with refined energies between − 18.70 and − 23.39 kcal/mol. On the other hand, chitosan demonstrated stronger binding with scores from − 5.85 to − 6.42 kcal/mol, and refined energies up to − 34.22 kcal/mol. The data in Table 6 demonstrated the interactions among the tested compounds and target proteins. Methyl gallate formed H-bond interactions with ASP362 (OD1, distance 3.2 Å, − 1.2 kcal/mol) and ALA169 (O, distance 3.2 Å, − 2.3 kcal/mol). Chitosan interacted with ASP223 (OD2, distance 3.1 Å, − 0.8 kcal/mol) and HIS323 (NE2, distance 2.8 Å, − 3.2 kcal/mol). 2D and 3D ligand–protein interaction diagrams (Fig. 6) confirmed stable binding at the urease active site. Methyl gallate and chitosan both exhibited inhibitory potential against *H. pylori* urease, a critical enzyme for bacterial survival in acidic gastric environments.

**Table 6:** Interaction of methyl gallate and chitosan with structure of *H. pylori* (PDB ID: 6ZJA)

Mol	Ligand	Receptor	Interaction	Distance	E (kcal/mol)
Methyl gallate	O 3	OD1 ASP 362 (B)	H-donor	3.2	− 1.2
	O 6	O ALA 169 (B)	H-donor	3.2	− 2.3
Chitosan	O 16	OD2 ASP 223 (B)	H-donor	3.1	− 0.8
	O 1	NE2 HIS 323 (B)	H-acceptor	2.9	− 3.2

The observed docking scores are consistent with previously reported natural urease inhibitors. The higher affinity of chitosan compared to methyl gallate can be attributed to its polycationic nature, enabling strong electrostatic and hydrogen bonding interactions with acidic residues such as ASP223. Similar findings have been reported for chitosan derivatives against bacterial enzymes, demonstrating antimicrobial and anti-biofilm properties. Methyl gallate’s moderate binding affinity aligns with experimental studies showing phenolic gallates as effective urease inhibitors. Its interactions with ALA169 and ASP362 highlight its potential to interfere with the catalytic mechanism. On the other hand, chitosan’s interaction with HIS323, a residue critical for urease stability, indicates possible strong inhibition. The findings suggest that while Chitosan may exhibit stronger direct inhibitory potential due to its binding, Methyl gallate could play a significant role in a multi-pronged approach to combat *H. pylori*. The concept of combining chitosan with gallate compounds, such as in chitosan gallate, has already been explored to enhance activity and solubility, highlighting a promising avenue for future drug development. The relevance of molecular docking in identifying and validating such interactions is underscored by its widespread use in drug discovery (Bazaid et al. 2025). It allows researchers to predict potential drug candidates and elucidate their mechanisms of action at a molecular level, supporting experimental validation through in vitro and in vivo studies.

## Conclusion

This study demonstrated that encapsulation of ASSE within CS-NPs significantly enhanced its in vitro therapeutic potential. The phytochemical profiling confirmed the abundance of phenolic acids and flavonoids, with methyl gallate as the dominant constituent. Successful encapsulation was validated through FTIR, indicating strong hydrogen bonding and electrostatic interactions between ASSE and CS-NPs. The formulated ASSE@CS-NPs exhibited remarkable in vitro biological activity across all tested assays. Antimicrobial evaluation revealed superior anti-*H. pylori* effects, with the largest inhibition zone (23.7 mm) and lowest MIC/MBC (15.62 µg/mL), surpassing both ASSE and CS-NPs alone. In antioxidant assays, ASSE@CS-NPs displayed notable free-radical scavenging an IC_50_ of 3 µg/mL, upon using DPPH and 15 ± 0.1 µg/mL, upon using ABTS testing closely approaching ascorbic acid. Furthermore, cytotoxicity assays against HCT116 cells demonstrated strong anticancer activity, achieving > 80% inhibition at 62.5 µg/mL and an IC_50_ of 23 µg/mL. Overall, ASSE@CS-NPs represent a promising nanoplatform for enhancing antimicrobial, antioxidant, and anticancer applications. This docking study demonstrates that both methyl gallate and chitosan bind effectively to the active site of *H. pylori* urease (6ZJA), with chitosan showing stronger binding affinity. The results support methyl gallate as a promising small-molecule inhibitor and chitosan as a macromolecular anti-*H. pylori* candidate, with potential synergistic applications in gastric infection therapy. Future research is advised to: (1) Increase production using economical and environmentally friendly techniques, which will be essential for industrial applications. (2) To confirm enhanced bioavailability, antioxidant, antibacterial, and therapeutic benefits seen in vitro, in vivo studies are necessary. (3) Creating targeted and stimuli-responsive chitosan nanocarriers that allow for site-specific distribution in the stomach or other organs.

### Limitations of the study

The shelf life of nanoparticles may be compromised by aggregation or loss of structural integrity during storage. Optimization and large-scale production are further hindered by inadequate encapsulation mechanism characterization. Moreover, the necessity for consistent and thorough research methodologies is highlighted by the lack of in vivo validation and possible variation in chitosan sources, which pose concerns about bioavailability, safety, and regulatory approval.

## Supplementary Information

Below is the link to the electronic supplementary material.

Supplementary Material 1

## Data Availability

The results from the present investigation are available from the corresponding author upon reasonable appeal.

## References

[CR1] Abdel Ghany T, Hakamy OM (2014) *Juniperus procera* as food safe additive, their antioxidant, anticancer and antimicrobial activity against some food-borne bacteria. J Biol Chem Res 31:668–677

[CR2] Abdel-Farid IB, Sheded MG, Mohamed EA (2014) Metabolomic profiling and antioxidant activity of some *Acacia* species. Saudi J Biol Sci 21:400–408. 10.1016/j.sjbs.2014.03.005 25313274 10.1016/j.sjbs.2014.03.005 PMC4191618

[CR3] Abd-ElGawad A, El-Amier Y (2015) Allelopathy and potential impact of invasive *Acacia saligna* (Labill.) Wendl. on plant diversity in the Nile Delta coast of Egypt. Int J Environ Res 9:923–932

[CR4] Abdelghany TM, Ganash M, Alawlaqi MM (2019) Antioxidant, antitumor, antimicrobial activities evaluation of *Musa paradisiaca* L. pseudostem exudate cultivated in Saudi Arabia. BioNanoScience 9:172–178. 10.1007/s12668-018-0580-x

[CR5] Abdelghany TM, Hassan MM, El-Naggar MA, Abd El-Mongy M (2020) GC/MS analysis of *Juniperus procera* extract and its activity with silver nanoparticles against *Aspergillus flavus* growth and aflatoxins production. Biotechnol Rep 27:e00496. 10.1016/j.btre.2020.e00496 10.1016/j.btre.2020.e00496 PMC7327896 32637346

[CR6] Adiamo OQ, Netzel ME, Hoffman LC, Sultanbawa Y (2020) Acacia seed proteins: low or high quality? A comprehensive review. Compr Rev Food Sci Food Saf 19:21–43 33319524 10.1111/1541-4337.12508

[CR7] Ahmed AZ, Satyam SM, Shetty P, and D'Souza MR (2021) Methyl Gallate Attenuates Doxorubicin-Induced Cardiotoxicity in Rats by Suppressing Oxidative Stress. Science (Cairo) 6694340. 10.1155/2021/6694340 10.1155/2021/6694340 PMC7822703 33510932

[CR8] Alajmi MF, Alam P, Alqasoumi SI, Ali Siddiqui N, Basudan OA, Hussain A, Mabood Husain F, Ali Khan A (2017) Comparative anticancer and antimicrobial activity of aerial parts of *Acacia salicina, Acacia laeta, Acacia hamulosa* and *Acacia tortilis* grown in Saudi Arabia. Saudi Pharm J 25:1248–1252 29204075 10.1016/j.jsps.2017.09.010 PMC5688228

[CR9] Alawlaqi MM, Al-Rajhi AMH, Abdelghany TM, Ganash M, Moawad H (2023) Evaluation of biomedical applications for linseed extract: antimicrobial, antioxidant, anti-diabetic, and anti-inflammatory activities in vitro. J Funct Biomater 14(6):300. 10.3390/jfb14060300 37367264 10.3390/jfb14060300 PMC10299631

[CR10] Al-Huqail AA, Behiry SI, Salem MZM, Ali HM, Siddiqui MH, Salem AZM (2019) Antifungal, antibacterial, and antioxidant activities of *Acacia saligna* (Labill.) H. L. Wendl. flower extract: HPLC analysis of phenolic and flavonoid compounds. Molecules 24:700 30781352 10.3390/molecules24040700 PMC6412425

[CR11] Al-Rajhi AMH, Abdelghany TM (2023a) In vitro repress of breast cancer by bio-product of edible *Pleurotus ostreatus* loaded with chitosan nanoparticles. Appl Biol Chem 66:33. 10.1186/s13765-023-00788-0

[CR12] Al-Rajhi AMH, Abdelghany TM (2023b) Nanoemulsions of some edible oils and their antimicrobial, antioxidant, and anti-hemolytic activities. BioResources 18(1):1465–1481. 10.15376/biores.18.1.1465-1481

[CR13] Al-Rajhi AMH, Qanash H, Almashjary MN, Hazzazi MS, Felemban HR, Abdelghany TM (2023a) Anti-*Helicobacter pylori*, antioxidant, antidiabetic, and anti-Alzheimer’s activities of laurel leaf extract treated by moist heat and molecular docking of its flavonoid constituent, naringenin, against acetylcholinesterase and butyrylcholinesterase. Life 13(7):1512. 10.3390/life13071512 37511887 10.3390/life13071512 PMC10382016

[CR14] Al-Rajhi AMH, Qanash H, Bazaid AS, Binsaleh NK, Abdelghany TM (2023b) Pharmacological evaluation of *Acacia nilotica* flower extract against *Helicobacter pylori* and human hepatocellular carcinoma in vitro and in silico. J Funct Biomater 14(4):237. 10.3390/jfb14040237 37103327 10.3390/jfb14040237 PMC10143343

[CR15] Al-Rajhi AMH, Abdelghany TM, Almuhayawi MS, Alruhaili MH, Saddiq AA, Baghdadi AM, Selim S (2024) Effect of ozonation on the phytochemicals of black seed oil and its anti-microbial, anti-oxidant, anti-inflammatory, and anti-neoplastic activities in vitro. Sci Rep 14:30445. 10.1038/s41598-024-81157-9 39663384 10.1038/s41598-024-81157-9 PMC11634964

[CR16] Alsalamah SA, Alghonaim MI, Abdelghany TM et al (2025a) Effect of UV-C radiation on chemical profile and pharmaceutical application in vitro of *Aloe vera* oil. AMB Express 15:83. 10.1186/s13568-025-01884-8 40439784 10.1186/s13568-025-01884-8 PMC12122980

[CR17] Alsalamah SA, Alghonaim MI, Mohammad AM et al (2025b) Ozone-modified properties of pumpkin seed oil as anti-*H. pylori*, anticancer, anti-diabetic and anti-obesity agent. Sci Rep 15:25959. 10.1038/s41598-025-11123-6 40676077 10.1038/s41598-025-11123-6 PMC12271415

[CR18] Alsolami A, Bazaid AS, Alshammari MA et al (2025) Ecofriendly fabrication of natural jojoba nanoemulsion and chitosan/jojoba nanoemulsion with studying the antimicrobial, anti-biofilm, and anti-diabetic activities in vitro. Biomass Conv Bioref 15:1283–1294. 10.1007/s13399-023-05162-0

[CR19] Bakri MM, Alghonaim MI, Alsalamah SA et al (2024) Impact of moist heat on phytochemical constituents, anti-*Helicobacter pylori*, antioxidant, anti-diabetic, hemolytic and healing properties of rosemary plant extract in vitro. Waste Biomass Valor 15:4965–4979. 10.1007/s12649-024-02490-8

[CR20] Bazaid AS, Binsaleh NK, Barnawi H et al (2025) Unveiling the in vitro activity of extracted *Euphorbia trigona* via supercritical fluid extraction against pathogenic yeasts, obesity, cancer, and its wound healing properties. Bioresour Bioprocess 12:28. 10.1186/s40643-025-00855-y 40183897 10.1186/s40643-025-00855-y PMC11971087

[CR21] Bhandari A, Crowe SE (2012) *Helicobacter pylori* in gastric malignancies. Curr Gastroenterol Rep 14:489–496. 10.1007/s11894-012-0296-y 23054813 10.1007/s11894-012-0296-y

[CR22] Binsaleh NK, Bazaid AS, Barnawi H et al (2025) Chemical characterization, anticancer, antioxidant and anti-obesity activities with molecular docking studies of *Pleurotus ostreatus* biomass exposed to moist heat. Food Measure 19:2476–2495. 10.1007/s11694-025-03125-9

[CR23] Castro-Muñoz R, Cabezas R (2026) From non-porous to highly porous homogeneous and heterogeneous structures: the evolving role of deep eutectic solvents in customizing chitosan-based materials—a review. Adv Colloid Interface Sci 349:103756. 10.1016/j.cis.2025.103756 41435502 10.1016/j.cis.2025.103756

[CR24] Castro-Muñoz R, René Cabezas R, Plata-Gryl M (2024) Mangiferin: a comprehensive review on its extraction, purification and uses in food systems. Adv Colloid Interface Sci 329:103188. 10.1016/j.cis.2024.103188 38761602 10.1016/j.cis.2024.103188

[CR25] Chang S-H, Hsieh P-L, Tsai G-J (2020) Chitosan inhibits *Helicobacter pylori* growth and urease production and prevents its infection of human gastric carcinoma cells. Mar Drugs 18(11):542. 10.3390/md18110542 33138146 10.3390/md18110542 PMC7692773

[CR26] Chang PK, Tsai MF, Huang CY, Lee CL, Lin C, Shieh CJ, Kuo C-H (2021) Chitosan-based anti-oxidation delivery nano-platform: applications in the encapsulation of DHA-enriched fish oil. Mar Drugs 19(8):470. 10.3390/md19080470 34436309 10.3390/md19080470 PMC8400499

[CR27] Correa LB, Seito LN, Manchope MF, Verri WA, Cunha TM, Henriques MG et al (2020) Methyl gallate attenuates inflammation induced by Toll-like receptor ligands by inhibiting MAPK and NF-Κb signaling pathways. Inflamm Res 69(12):1257–1270. 10.1007/s00011-020-01407-0 33037469 10.1007/s00011-020-01407-0

[CR28] da Silva SB, Amorim M, Fonte P, Madureira R, Ferreira D, Pintado M et al (2015) Natural extracts into chitosan nanocarriers for rosmarinic acid drug delivery. Pharm Biol 53:642–652. 10.3109/13880209.2014.935949 25489634 10.3109/13880209.2014.935949

[CR29] Devi CS, Tarafder A, Shishodiya E, Mohanasrinivasan V (2015) Encapsulation of staphylokinase and *Leucasaspera* plant extracts using chitosan nanoparticles. Int J Pharmtech Res 7:654–661

[CR30] Dunn BE, Cohen H, Blaser MJ (1997) Helicobacter pylori. Clin Microbiol Rev 10:720–741. 10.1128/CMR.10.4.720 9336670 10.1128/cmr.10.4.720 PMC172942

[CR31] Elmi A, Spina R, Risler A, Philippot S, Mérito A, Duval RE, Abdoul-latif FM, Laurain-Mattar D (2020) Evaluation of antioxidant and antibacterial activities, cytotoxicity of *Acacia seyal* Del bark extracts and isolated compounds. Molecules 25(10):2392. 10.3390/molecules25102392 32455580 10.3390/molecules25102392 PMC7288156

[CR32] Eranda DHU, Chaijan M, Panpipat W, Karnjanapratum S, Cerqueira MA, Castro-Muñoz R (2024) Gelatin-chitosan interactions in edible films and coatings doped with plant extracts for biopreservation of fresh tuna fish products: a review. Int J Biol Macromol 280(Pt 2):135661. 10.1016/j.ijbiomac.2024.135661 39299417 10.1016/j.ijbiomac.2024.135661

[CR33] Ferreyra-Suarez D, Paredes-Vargas L, Jafari SM, García-Depraect O, Castro-Muñoz R (2024) Extraction pathways and purification strategies towards carminic acid as natural-based food colorant: a comprehensive review. Adv Colloid Interface Sci 323:103052. 10.1016/j.cis.2023.103052 38086153 10.1016/j.cis.2023.103052

[CR34] Foyzun T, Mahmud AA, Ahammed MS, Manik MIN, Hasan MK, Islam KMM, Lopa SS, Al-Amin MY, Biswas K, Afrin MR et al (2022) Polyphenolics with strong antioxidant activity from *Acacia nilotica* ameliorate some biochemical signs of Arsenic-induced neurotoxicity and oxidative stress in mice. Molecules 27:1037. 10.3390/molecules27031037 35164302 10.3390/molecules27031037 PMC8840196

[CR35] Hussen EM, Endalew SA (2023) In vitro antioxidant and free-radical scavenging activities of polar leaf extracts of *Vernonia amygdalina*. BMC Complement Med Ther 23:146. 10.1186/s12906-023-03923-y 37143058 10.1186/s12906-023-03923-y PMC10157976

[CR36] Kaur P, Arora S, Singh R (2022) Isolation, characterization and biological activities of betulin from *Acacia nilotica* bark. Sci Rep 12:9370. 10.1038/s41598-022-13338-3 35672366 10.1038/s41598-022-13338-3 PMC9174266

[CR37] Liang H, Chen Z, Yang R, Huang Q, Chen H, Chen W, Zhang Y (2022) Methyl gallate suppresses the migration, invasion, and epithelial-mesenchymal transition of hepatocellular carcinoma cells via the AMPK/NF-κB signaling pathway in vitro and in vivo. Front Pharmacol 13:894285. 10.3389/fphar.2022.894285 35770085 10.3389/fphar.2022.894285 PMC9234279

[CR38] Magnini RD, Hilou A, Millogo-Koné H, Compaore S, Pagès JM, Davin-Regli A (2020) A review on ethnobotanical uses, biological activities, and phytochemical aspects of *Acacia senegal* (L.) Willd. and *Acacia seyal* Delile. (Fabaceae). Int J Plant Sci Hor 2:32–55. 10.36811/ijpsh.2020.110023

[CR39] Mashraqi A (2023) Induction role of chitosan nanoparticles to *Anethum graveolens* extract against food-borne bacteria, oxidant, and diabetic activities in vitro. Front Microbiol 14:1209524. 10.3389/fmicb.2023.1209524 37469433 10.3389/fmicb.2023.1209524 PMC10352794

[CR40] Negi A, Kesari KK (2022) Chitosan nanoparticle encapsulation of antibacterial essential oils. Micromachines 13:1265. 10.3390/mi13081265 36014186 10.3390/mi13081265 PMC9415589

[CR41] Qanash H, Bazaid AS, Aldarhami A, Alharbi B, Almashjary MN, Hazzazi MS, Felemban HR, Abdelghany TM (2023a) Phytochemical characterization and efficacy of *Artemisia judaica* extract loaded chitosan nanoparticles as inhibitors of cancer proliferation and microbial growth. Polymers 15:391. 10.3390/polym15020391 36679271 10.3390/polym15020391 PMC9865519

[CR42] Qanash H, Al-Rajhi AMH, Almashjary MN et al (2023b) Inhibitory potential of rutin and rutin nano-crystals against *Helicobacter pylori*, colon cancer, hemolysis and butyrylcholinesterase in vitro and in silico. Appl Biol Chem 66:79. 10.1186/s13765-023-00832-z

[CR43] Subhaswaraj P, Sowmya M, Jobina R, Sudharshan SJ, Dyavaiah M, Siddhardha B (2017) Determination of antioxidant potential of *Acacia nilotica* leaf extract in oxidative stress response system of *Saccharomyces cerevisiae*. J Sci Food Agric 97:5247–5253. 10.1002/jsfa.8409 28474422 10.1002/jsfa.8409

[CR44] Surendhiran D, Li C, Cui H, Lin L (2020) Fabrication of high stability active nanofibers encapsulated with pomegranate peel extract using chitosan/PEO for meat preservation. Food Packag Shelf Life 23:100439

[CR45] Sze Kwan L, Tzi Bun N (2010) Acafusin, a dimeric antifungal protein from *Acacia confusa* seeds. Protein Pept Lett 17:817–822 19958279 10.2174/092986610791306643

[CR46] Thotathil V, Rizk HH, Fakrooh A, Sreerama L (2022) Phytochemical analysis of *Acacia ehrenbergiana* (Hayne) grown in Qatar: identification of active ingredients and their biological activities. Molecules 27(19):6400. 10.3390/molecules27196400 36234937 10.3390/molecules27196400 PMC9571875

[CR47] Yahya R, Al-Rajhi AMH, Alzaid SZ, Al Abboud MA, Almuhayawi MS, Al Jaouni SK et al (2022) Molecular docking and efficacy of *Aloe vera* gel based on chitosan nanoparticles against *Helicobacter pylori* and its antioxidant and anti-inflammatory activities. Polymers 14:2994. 10.3390/polym14152994 35893958 10.3390/polym14152994 PMC9330094

