# Supplementary material for: Chitosan-mediated nano-bioprocessing of Acacia seyal seed extract for enhanced antineoplastic, anti-Helicobacter pylori and antioxidant performance
Source: Bioresour Bioprocess. 2026 Apr 2;13(1):46. doi: 10.1186/s40643-026-01031-6 (PMC13047003; doi:10.1186/s40643-026-01031-6)
Supplement: Supplementary file 1 — Supplementary Material 1 [file 40643_2026_1031_MOESM1_ESM.docx]

**Suppletory 1. Docking scores and energies of methyl gallate and chitosan with structure of H. pylori (PDB ID: 6ZJA).**

| **Mol** | **S** | **rmsd_refine** | **E_conf** | | **E_place** | **E_score1** | **E_refine** | **E_score2** |
| --- | --- | --- | --- | --- | --- | --- | --- | --- |
| Methyl gallate | -5.45 | 0.82 | -3.44 | | -62.94 | -11.01 | -21.33 | -5.45 |
| Methyl gallate | -5.38 | 1.21 | -0.19 | | -52.42 | -10.51 | -18.70 | -5.38 |
| Methyl gallate | -5.33 | 1.38 | -4.18 | | -76.39 | -12.57 | -23.39 | -5.33 |
| Methyl gallate | -5.33 | 1.94 | -4.38 | | -54.57 | -10.79 | -21.63 | -5.33 |
| Methyl gallate | -5.25 | 1.19 | -4.03 | | -66.88 | -11.14 | -22.06 | -5.25 |
| Chitosan | -6.42 | 2.70 | | -284.11 | -100.84 | -8.03 | -29.26 | -6.42 |
| Chitosan | -6.09 | 2.96 | | -261.85 | -95.02 | -10.24 | -22.76 | -6.09 |
| Chitosan | -6.05 | 1.66 | | -252.07 | -86.58 | -7.98 | -26.45 | -6.05 |
| Chitosan | -6.04 | 1.96 | | -250.18 | -67.03 | -9.53 | -34.23 | -6.04 |
| Chitosan | -5.85 | 2.15 | | -262.22 | -125.19 | -8.93 | -22.46 | -5.85 |
